# In vivo spectroscopy and machine learning for the early detection and classification of different stresses in apple trees

**DOI:** 10.1038/s41598-023-42428-z

**Published:** 2023-09-22

**Authors:** Ulrich E. Prechsl, Abraham Mejia-Aguilar, Cameron B. Cullinan

**Affiliations:** 1 Laimburg Research Centre, Laimburg 6, 39040 Auer, South Tyrol Italy; 2 Eurac Research, Drususallee 1/Viale Druso 1, 39100 Bolzano, South Tyrol Italy; 3 Faculty of Agricultural, Environmental and Food Sciences, Free University of Bolzano, Piazza Università 1, 39100 Bolzano, South Tyrol Italy

**Keywords:** Plant stress responses, Abiotic, Biotic, Flooding, Near-infrared spectroscopy

## Abstract

The use of in vivo spectroscopy to detect plant stress in its early stages has the potential to enhance food safety and reduce the need for plant protection products. However, differentiating between various stress types before symptoms appear remains poorly studied. In this study, we investigated the potential of Vis–NIR spectroscopy to differentiate between stress types in apple trees (*Malus x domestica* Borkh.) exposed to apple scab, waterlogging, and herbicides in a greenhouse. Using a spectroradiometer, we collected spectral signatures of leaves still attached to the tree and utilized machine learning techniques to develop predictive models for detecting stress presence and classifying stress type as early as 1–5 days after exposure. Our findings suggest that changes in spectral reflectance at multiple regions accurately differentiate various types of plant stress on apple trees. Our models were highly accurate (accuracies between 0.94 and 1) when detecting the general presence of stress at an early stage. The wavelengths important for classification relate to photosynthesis via pigment functioning (684 nm) and leaf water (~ 1800–1900 nm), which may be associated with altered gas exchange as a short-term stress response. Overall, our study demonstrates the potential of spectral technology and machine learning for early diagnosis of plant stress, which could lead to reduced environmental burden through optimizing resource utilization in agriculture.

## Introduction

Plant stress, i.e. “*external conditions that adversely affect growth, development, or productivity*”^1^, is a significant factor that limits crop yield and can have far-reaching consequences on food security and the economy. Therefore, understanding the underlying physiological mechanisms and developing effective stress management strategies are crucial for sustainable agriculture. It is estimated that abiotic stress due to harsh environmental conditions such as drought and salinity reduce the average productivity by more than 50%^2^. Similarly, biotic stresses, caused by organisms such as pests and pathogens, are responsible for high losses up to 40% of the main global crops^3^. By definition “stress factors” subsequently lead to different “stress responses” of the plant^4^. Plants can respond to biotic and abiotic stresses with different short and long-term resistance mechanisms, ranging from molecular physiological levels (such as the jasmonate and salicylic acid signalling, phytoalexines and heat-shock proteins) to the morphological level (cell structure, growth)^1,5–11^. Generic stress responses at an advanced stage are different types of chlorosis (pigment degradation) and necrosis which are typically used for visual diagnosis purposes^12,13^. However, visible stress symptoms are typically associated with high ‘metabolic costs’, damage and reduction in assimilates and crop yield as a consequence^13^. In order to efficiently counteract productivity loss, early detection of plant stresses when symptoms are often not yet visible is crucial.

The detection and quantification of emitted or reflected electromagnetic radiation, particularly that in the visible and infrared regions, by plant surfaces, has become an established and powerful method to analyse plant stress and various plant properties in a non-destructive way^14–17^. One of the most prominent applications is the normalized difference vegetation index (NDVI). Originally developed for the remote detection of plant stress, particularly drought, the NDVI, has also been applied to other abiotic stresses, such as salinity^18,19^. Subsequently, the relationship between leaf biochemistry, physiology and cellular structure and leaf optical properties, especially those related to full range VIS -NIR hyperspectral information, were investigated and modelled^20,21^. This had promising implications for the use of the technology for the detection of stress in plants and indeed, spectral sensing has since been applied over the last decades to detect a wide range of different biotic plant stresses: viruses^22^, bacteria^23^, phytoplasma^24,25^, fungal and bacterial diseases^26,27^ and insect pests^28^.

For permanent crops, such as apple orchards (*Malus x domestica* Borkh.), early detection is especially important because management practices can have cumulative effects on long term (~ 20 years) production and financial amortization. Furthermore, permanent crops cannot benefit from crop rotation and consequently experience higher pest and disease pressure.

The management of stress, especially those involving agrochemical use, can have unintended and undesirable consequences on the environment as well as ecosystem and human health^29–33^. Early detection of stress can limit the consequences thereof, not only because the agrochemical requirements of plants experiencing mild stress are lower than those experiencing more severe stress, but also by preventing the progression of stress, especially in cases of infectious pests and diseases. Furthermore, because spectral sensing methods have the potential to detect stress at fine (in this case, individual plant) scales, spectral based methods offer a means by which management practices can be made more site- and time-specific^34^. Hence, detecting plant stress at an early stage has the potential to (a) improve economic efficiency and (b) decrease agrochemical use thereby enhancing food safety while limiting negative impacts of agriculture on the environment.

Apple trees have been the focus of several studies utilizing spectral sensing methods to detect various plant diseases^26,35,36^. Most of these studies have focused on the detection of single diseases. However, the differentiation by means of spectral sensing among apple trees experiencing different stress types in the pre-symptomatic phases has been little investigated. This is important regarding the development of future field application tools, as in real orchard settings, various types of stress can occur simultaneously, often with different stresses causing similar symptoms. The aim of this study was, therefore, to examine the potential of in vivo leaf spectroscopy to distinguish different types of stress. Our research questions were as follows:

1. Is it possible to distinguish different types of stresses in the pre-symptomatic stage by means of spectrum analysis?
2. Is there a consistent (spectral) stress signal of apple trees, independent of the stress type?
3. Which are the key spectral regions for differentiation and are they physiologically plausible?

To address these questions, we conducted a greenhouse experiment in which we exposed small apple trees to different types of stress with subsequent measurement with a field spectroradiometer. The generated spectral data was analysed through the use of machine learning techniques.

## Results

### Mean spectral signature of apple leaves

**Figure 1:**
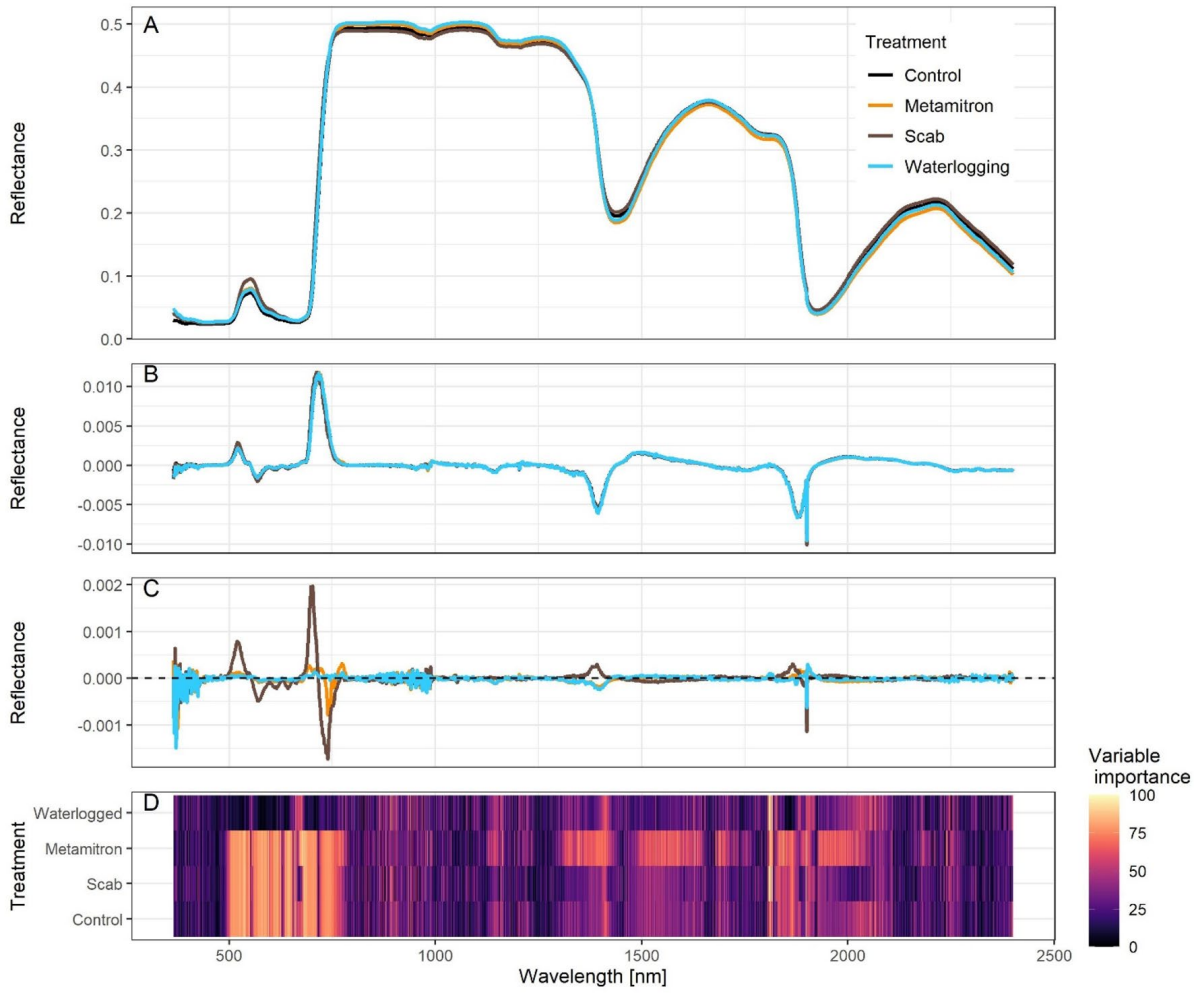
Spectral response (reflectance) of 3-year-old ‘Golden Delicious’ apple trees that were exposed to different types of stress (herbicide Metamitron, apple scab and waterlogging, n = 5). Panel (**A**) shows the mean spectral signature for each stress treatment (n = 50) and the control. Panel (**B**) presents the means for each treatment of the first derivative of the spectra. Panel (**C**) displays the delta plot highlighting the differences in the first derivatives between the stress treatments and the control. Panel (**D**) shows the variable importance (determined by ROC curve analysis) in the Random Forest model trained on the first derivative for classifying the four treatments.

The spectra obtained in our study revealed the characteristic broadband peaks and valleys of the spectral signature (reflectance) due to absorption by chlorophyll and water. The reflectance was found to be low in the visible range (300–700 nm), at 1400 nm and at 1900 nm. The coefficient of variation (CV) for the measured replicates ranged between 7.1 and 8.7, depending on the treatment. (see supplementary Fig. S1). The differences between the mean spectra of each of the four treatment groups were found to be slight (Fig. 1A). The only exception was the “Scab” treatment, which showed a higher reflectance at ~ 550 nm.

Upon analysing the transformed data (1st derivative; Fig. 1B), we observed very subtle differences, which became more distinct in the delta plot (Fig. 1C; treatment-control). The most pronounced visible differences were observed at around 500 nm, 700 nm, 1400 nm and 1900 nm. The region to the left of the spectrum (starting at 365 nm) exhibited relatively high noise.

### Principal component analysis

**Figure 2:**
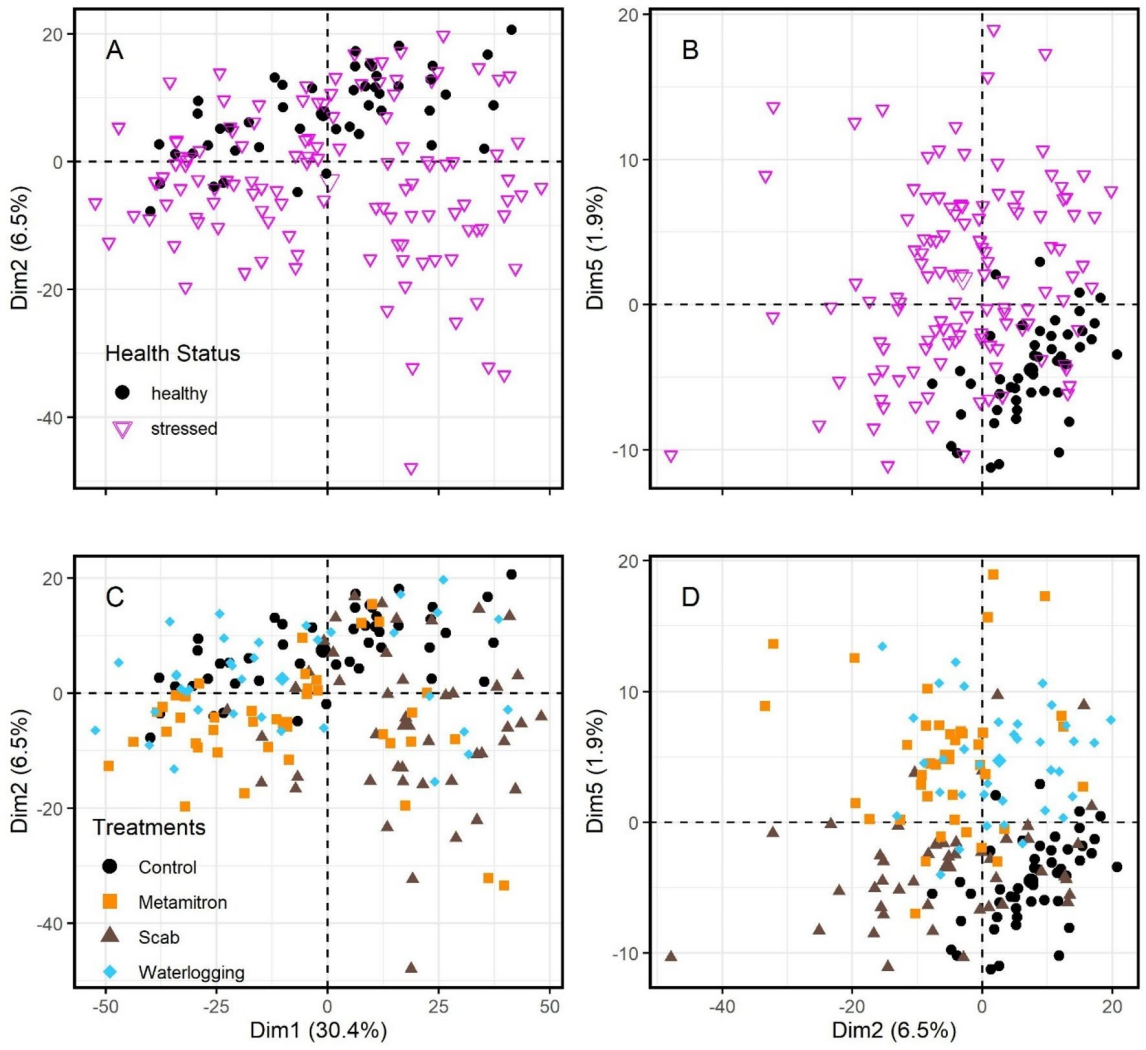
Principal component analysis (PCA) of spectral signatures of four treatments. The PCA is based on the first derivative (as seen in Fig. 1B) and displays the first, second, and fifth dimensions. Panels (**A**) and (**C**) and panels (**B**) and (**D**), display the same data and dimensions but with different labels, indicating the specific and general models. In the upper panels, the treatments of “Metamitron” (orange rectangles), “Scab” (brown triangles), and “Waterlogging” (light blue dots) have been grouped together as “stressed” (pink triangles). The “healthy” corresponds to “control” and is given by the black dots.

PCA (Principal Component Analysis) is a widely used technique in spectral analysis to identify patterns, trends and separate signals from different physical processes. We considered the first five components, explaining 46.08% of the variance (Table 1, Fig. 2). The first four dimensions of the PCA did not show good clustering of the classes, suggesting that the class separation is not strongly reflected in these components. This may indicate that factors other than stress are driving the variance in the data and obscuring the class distinctions. The fifth dimension (1.9% of the variance), when combined with the second dimension (6.9%), showed acceptable clustering of the classes, for both the specific (treatments) and the general (status) differentiation. This suggests that the class separation is better captured by these components. When looking at the loadings of the components, the spectral regions around 660 nm and 1880 nm were found to be strongly correlated with the second PC and those around 1810 nm with the fifth PC. This indicates that these regions may be significant factors in the class separation captured by these components (see Table1). The first dimension, which explained 30.4% of the variance, was found to be strongly correlated with the region around 1300 nm, but it showed insufficient separation of the classes. The region around 1300 nm in the leaf reflectance spectrum is strongly influenced by the absorption of water in the leaf, which leads to a decrease in reflectance in this region and one of the typical “valleys”. Our delta-plot reveals slight but distinct differences between the treatments in this region.

**Table 1:** Important wavelengths for the classification of different stresses and stress in general. Presented are the overall top 10 important variables for the classification of specific stresses as well as stresses in general by random forest models, calculated as the impurity-corrected Gini importance, the variables with the top 10 highest correlation loadings with the first, second and fifth PCs (PCA) and the top 10 important variables specific to each of the treatments calculated by ROC curve analysis using the random forest model. Numbers refer to wavelengths of each variable in nm which are sorted in decreasing order of importance.

Method	Random forest (Gini)	Random forest (Gini)	PCA			ROC-curve class specific variable importance			
	Specific model	General stress model	Dimension (variance explained)			Control	Metamitron	Scab	Waterlogging
			1 (30.40%)	2 (6.50%)	5 (1.90%)				
Important variables (decreasing; wavelength [nm])	1811	1900	1391	1881	1813	684	684	1811	1811
	1817	1899	1390	1879	1814	536	536	684	1810
	1814	940	1389	1880	1812	596	679	1810	1817
	1900	894	1387	1882	1815	537	677	1817	1808
	684	663	1392	1884	1811	1900	596	536	1814
	1810	832	1388	662	1817	683	537	596	832
	1813	662	1386	665	1816	615	1900	537	1812
	1816	881	1385	661	1808	645	683	1808	1809
	919	2055	1383	660	1810	1899	615	1900	2055
	1899	944	1384	663	1818	612	1813	1814	892

### Training and evaluating different diagnosis models

We tested different supervised machine learning approaches: a Support Vector Machine (SVM), a Random Forest, and a Partial Least Squares-Discriminant Analysis (PLS-DA) model for multi-class classification to distinguish, by means of their spectral signatures, the different types of stresses (treatments) in the pre-symptomatic stage. We assessed the performance of the models by means of the overall accuracy and Kappa metrics using the test data (see Table 2). The SVM model achieved an overall accuracy of 1, while the Random Forest model had an overall accuracy of 0.84 (Table 2). The PLS model also had an overall accuracy of 1, with the number of components (latent variables) determined via the validation process to be 11. The general differentiation between “healthy” (control) and “stressed” plants (independent of the type of stress) performed even better (where possible; Table 3). The overall accuracies were 1, 0.94 and 1 for the SVM, Random Forest and PLS, respectively.

**Table 2:** Overall and class-specific test set metrics for SVM, PLS-DA and random forest models trained to predict individual stresses using the full derivative spectra.

	Sensitivity/recall	Specificity	Precision	F1	Balanced accuracy
SVM					
	Accuracy (overall) = 1.0000		Kappa (overall) = 1.0000		
Control	1.0000	1.0000	1.0000	1.0000	1.0000
Scab	1.0000	1.0000	1.0000	1.0000	1.0000
Metamitron	1.0000	1.0000	1.0000	1.0000	1.0000
Water logging	1.0000	1.0000	1.0000	1.0000	1.0000
PLS-DA					
LV = 11	Accuracy (overall) = 1.0000		Kappa (overall) = 1.0000		
Control	1.0000	1.0000	1.0000	1.0000	1.0000
Scab	1.0000	1.0000	1.0000	1.0000	1.0000
Metamitron	1.0000	1.0000	1.0000	1.0000	1.0000
Water logging	1.0000	1.0000	1.0000	1.0000	1.0000
Random forest					
	Accuracy (overall) = 0.8438		Kappa (overall) = 0.7849		
Control	1.0000	0.8571	0.7857	0.8800	0.9286
Scab	0.7143	0.9200	0.7143	0.7143	0.8171
Metamitron	0.7143	1.0000	1.0000	0.8333	0.8571
Water logging	0.8571	1.0000	1.0000	0.9231	0.9286

**Table 3:** Test set metrics for SVM, PLS-DA and random forest models trained to predict stress in general using full spectra and the top 10 most important wavelengths of first derivative spectra identified by the full random forest model.

	Accuracy (overall)	Kappa (overall)	Sensitivity/recall	Specificity	Precision	F1	Balanced accuracy
Full spectra							
SVM	1.000	1.000	1.000	1.000	1.000	1.000	1.000
PLS-DA (LV = 4)	1.000	1.000	1.000	1.000	1.000	1.000	1.000
Random forest	0.939	0.836	1.000	0.923	0.778	0.875	0.962
Selected wavelengths							
SVM	0.970	0.921	0.889	1.000	1.000	0.941	0.944
PLS-DA (LV = 1)	0.939	0.847	0.889	0.958	0.889	0.889	0.924
Random forest	0.939	0.836	0.778	1.000	1.000	0.875	0.889

Overfitting of our models was assessed by inspection of learning curves of the random forest and SVM models and of the calibration curves of the PLS-DA models (Supplementary Figs. S2 and S3). Its absence was further confirmed by high cross-validation (tenfold cross-validation with 3 repeats) overall accuracy which was largely insensitive to changes in hyperparameters in the cross-validation results, indicating the models generalize well to unseen data.

In summary, all models were able to identify and distinguish the stresses with high accuracy.

### Reduced models based on important variables

The identification of key features is integral to gaining insights into the underlying physiological mechanisms, as these features are likely to be closely associated with the relevant physiological processes. These features can subsequently be used to simplify the model, by removing irrelevant or redundant features. Moreover, due to reduced complexity, a simpler model is easier to understand, has faster computation, and the likelihood of overfitting is reduced.

#### Stress-specific models

For the multi-class classification, the Random Forest method identified wavelengths around 1800 nm and 1900 nm, as well around 684 nm as the most important wavelengths (see Table 1). The reduced models, based on the top 10 wavelength yielding accuracies of 0.72, 0.78, and 0.75 for the SVM, Random Forest, and PLS models, respectively (Table 4).

**Table 4:** Overall and class-specific test set metrics for SVM, PLS-DA and random forest models trained to predict individual stresses using the top 10 most important wavelengths of first derivative spectra identified by the full random forest model.

	Sensitivity/recall	Specificity	Precision	F1	Balanced accuracy
SVM					
	Accuracy (overall) = 0.7188		Kappa (overall) = 0.6211		
Control	0.7273	0.9048	0.8000	0.7619	0.8160
Scab	1.0000	0.8000	0.5833	0.7368	0.9000
Metamitron	0.2857	0.9600	0.6667	0.4000	0.6229
Water logging	0.8571	0.9600	0.8571	0.8571	0.9086
PLS-DA					
LV = 3	Accuracy (overall) = 0.7500		Kappa (overall) = 0.6614		
Control	0.8182	0.9048	0.8182	0.8182	0.8615
Scab	1.0000	0.9200	0.7778	0.8750	0.9600
Metamitron	0.2857	0.9600	0.6667	0.4000	0.6229
Water logging	0.8571	0.8800	0.6667	0.7500	0.8686
Random forest					
	Accuracy (overall) = 0.7813		Kappa (overall) = 0.7068		
Control	0.7273	0.9524	0.8889	0.8000	0.8398
Scab	1.0000	0.8800	0.7000	0.8235	0.9400
Metamitron	0.5714	0.9200	0.6667	0.6154	0.7457
Water logging	0.8571	0.9600	0.8571	0.8571	0.9086

To uncover the mechanisms behind the differentiation between our treatments, we also analysed class-specific variable importance using the ROC analysis and the full Random Forest model (see Table 1). The heat map in Fig. 1D displays the variable importance for all treatments and wavelengths. Generally, the region in the visible range (~ 500–750 nm) showed high importance for the control group as well as the scab and herbicide treatments. For all treatments the region between 1800 and 2000 nm seems highly important. Interestingly, the region < 500 nm shows almost no importance.

**Figure 3:**
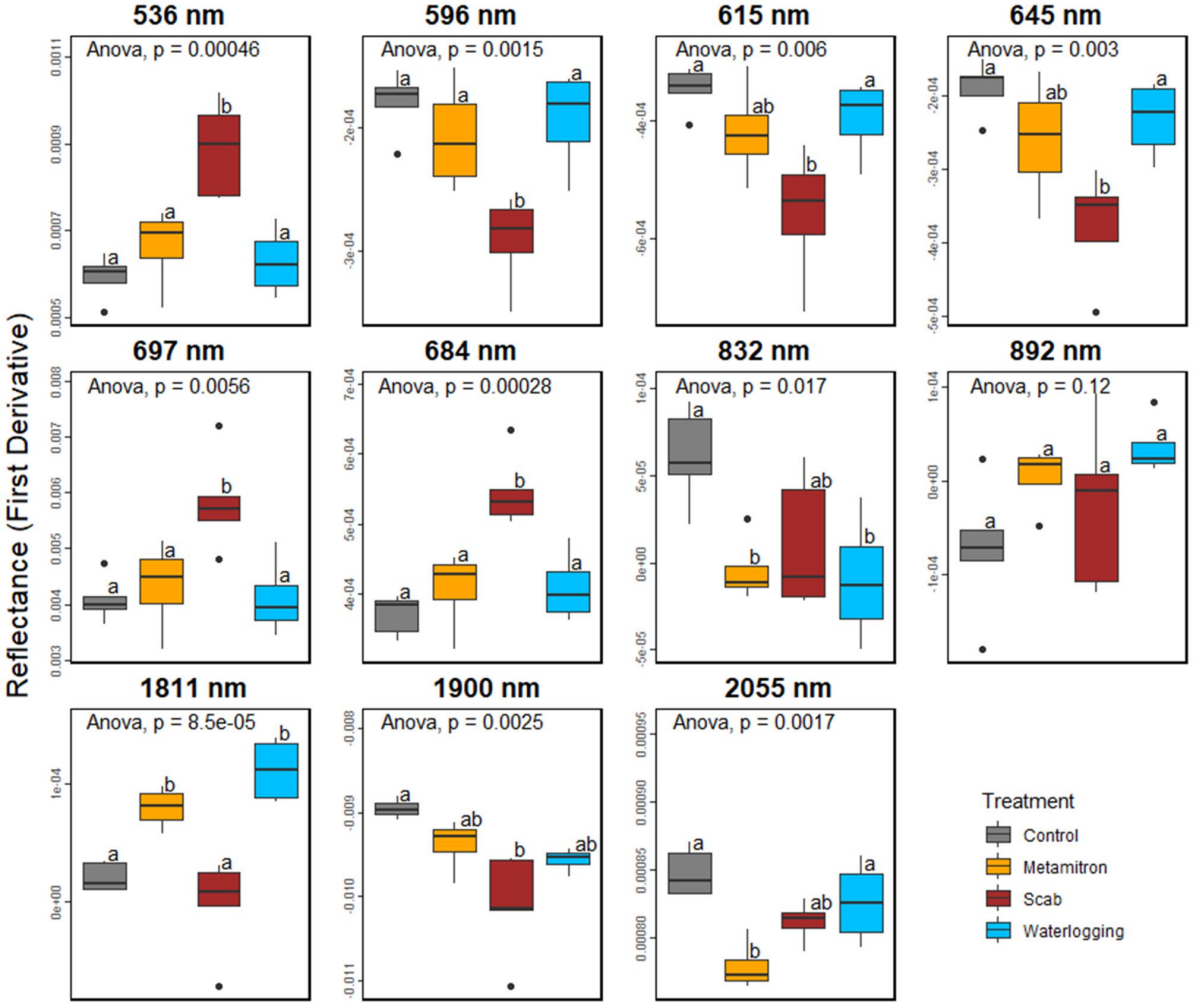
Selected wavelengths from various regions identified by random forest for differentiating treatments. The wavelengths were determined by class-specific variable importance, as shown in Table 4 and are based on the 1st derivative of the reflectance (see Fig. 1). Redundancies were removed considering wavelengths within a 10 nm range of the representative wavelength. The boxes display the distribution of spectral values at each wavelength, based on five replicates with 10 technical replicates each.

The wavelengths 1899 nm and 1900 nm were deemed important for all treatments except waterlogging. For the control treatment, wavelengths in the range of 536 nm to 684 nm were significant. The scab treatment had 536 nm, 595 nm, 684 nm and wavelengths between 1808 and 1817 nm as important. The herbicide treatment was found to have wavelengths between 537 and 684 nm as important. The waterlogging treatment was influenced by wavelengths ranging from 1808 to 1817 nm and 840 nm, as well as 832 nm, 892 nm and 2055 nm. To visually highlight the distinctions between treatments in specific areas, we plotted key wavelengths for regions that were identified as important (Fig. 3). It illustrates the presence of distinct differences across multiple regions.

#### General stress signal

To address the question of whether there is a general stress signal, regardless of the type of stress (treatment), we used variable importance of our Random Forest trained as a two-class model to distinguish between “healthy” and “stressed” samples. The top 10 most important wavelengths, other than for 1899 nm and 1900 nm, varied from the stress-specific model (Table 1). The other wavelengths were primarily around the border between near-infrared (NIR) and short-wave infrared (SWIR): 832 nm, 881 nm, 894 nm, 940 nm and 944 nm. Only two wavelengths in the visible region, 662 nm and 663 nm, were in the top 10 most important wavelengths.

## Discussion

### Models predict stresses with high accuracy

In this study, we aimed to assess the ability to detect and differentiate various types of stress in the pre-symptomatic stage through analysis of their spectral signatures. Early detection of plant stress is critical as it enables timely interventions to mitigate the impact and prevent further stress, ensuring plant health and productivity.

Our trained “stress signal” models displayed a high level of overall accuracy (full models between 0.94 and1, Table 3) and thus it was feasible (a) to detect the general presence of stress at a very early stage (1–5 days) and (b) that the models could also distinguish and classify the type of stress through spectral signatures very accurately (average overall accuracy: 0.95). The overall accuracy of the results was all the more remarkable given the absence of visible symptoms. Figure 3 and Fig. 1 D demonstrate that differentiation between the stress types is a result of the combined variations across multiple wavelengths in different regions, rather than in any single spectral region alone.

With the full dataset, the Partial Least Squares (PLS, 1) and Support Vector Machine (SVM, 1) models performed best, for both the specific and the general models. Both of these methods are particularly well suited to high dimensional data and have been used frequently in the past for the analysis of spectroscopic data on plant material^25,37–42^. This is because the internal regularization of SVMs and the reduction of the full data set into fewer variables done by PLS makes these models resilient to overfitting, as is demonstrated here by their high performance on the full data set^43,44^. Random forests, however, are not so implicitly regularized. As such, models built on datasets with a large number of features incur steep costs for variables with little explanatory power (as is often the case with spectroscopic data in which there is a high degree of correlation between each of the features). Utilizing PCA, as an alternative approach to PLS, can also help overcome issues of multicollinearity and reduce the dimensionality of the data and provide a means by which complex data can be more readily visualised. Upon conducting PCA analysis, we found that the first dimension did not exhibit strong clustering. Combining the second and fifth components resulted in notable clustering of the treatments (Fig. 2), with important wavelengths, according to the correlation loadings with these components, being similar to those determined by other methods. PCA, being an unsupervised method without any pre-defined labels, may be sensitive to variances that are not directly associated with the classes (stress types). As such, a minor component can explain a considerable amount of variation in the response variable, leading to weak clustering results. Therefore, it is important to consider multiple dimensions and to interpret the results in conjunction with other methods. Indeed, the theoretical advantage of PLS models over PCA, is that the first component automatically explains the most variance in the response variable and consequently fewer components are often required to yield satisfactory results^45^.

### Key wavelengths and possible underlying physiological bases thereof

The significance of the wavelengths around 1800 nm (1811 nm) and those around 1900 nm in particular, was highlighted in both the specific and general models (Fig. 3, Table 1), indicating that water absorption-related bands (such as 1900 nm) have a critical role in detecting and distinguishing stresses. The absorption at 1900 nm clearly distinguishes the healthy from the stressed plants. This could be attributed to stomatal regulation and photosynthesis alteration—two known general short-term responses to stress. The activation of ABA-dependent signaling pathways by oxidative stress induced by stressors such as drought, cold, salinity, and heat, regulate responses to various abiotic stresses via abscisic acid signaling^46–48^. As such, a general early response to abiotic stresses is often stomatal closure which is likely reflected in our measured changes in the “water valley”^49^.

The wavelengths, 677 nm, 679 nm and 684 nm are significant in the class-specific importances (Table 1). They fall within the red visible light spectrum and correspond to the peak of chlorophyll a absorption^50^. This implies that these stress treatments have the potential to impact the absorption related to chlorophyll a, the light reactions, and subsequently the functioning of photosynthesis. Notably, this effect appears to be particularly significant for the herbicide treatment, for which these wavelengths were among the most important (see Table 1). The herbicide Metamitron, a photosystem II inhibitor, induces oxidative stress inducing the excessive production of reactive oxygen species^51^. Plants respond to this type of oxidative stress, also caused by excess light stress, by activating various defence mechanisms, including dissipation of excess light energy through the Xanthophyll cycle. Interestingly, the classification of Metamitron-treated plants was influenced by the reflectance at 536 nm and 537 nm, wavelengths within the range used to develop the physiological reflectance (531 nm) index by Gamon et al.^52^, for the estimation of xanthophyll epoxidation states. This suggests that the Xanthophyll cycle may be involved in the response to Metamitron treatment.

Like the response to abiotic stress, the pathogen *Venturia inaequalis*, responsible for apple scab, induced a shift in the wavelength of the water valley (1900 nm) and wavelengths associated with pigment functioning (684 nm, 536 nm, 537 nm). The wavelength range of 510–550 nm was found to be positively correlated with the total pigment content of various tree species, especially carotenoids^53^. Carotenoids are part of the light harvesting complex and play a significant role in the quenching process, also to protect against oxidative stress^54^. While *Venturia inaequalis* is not known to directly affect stomatal aperture, it has been shown to secrete metabolites that attract water and solutes to the site of the lesions^55^. Previous research has demonstrated that infected apple trees have reduced leaf water potentials and higher water content, which could potentially explain the findings of our study (see Fig. 3: 1900 nm)^55,56^.

Delalieux et al. (2007) investigated the ability to differentiate between apple leaves infected with *V. inaequalis* (apple scab) and non-infected leaves via spectral methods. They found that good predictability could be achieved using supervised classification techniques^26^. They identified the spectral domains between 1350–1750 nm and 2200–2500 nm as the most important regions for distinguishing between infected and non-infected leaves. Furthermore, visible wavelengths around 650–700 nm were found to increase in importance three weeks after infection at a well-developed infection stage.

Finally, reflectance in the region between 800 and 990 nm is influenced by leaf properties such as thickness, density, and cellular structure, rather than plant pigments^21^. This region is often used as a normalization factor to improve correlations between wavelengths and biological constituents^53,57,58^. The importance of these NIR wavelengths as features may be related to their ability to correct for these effects, or to differences in cellular structure caused by stresses themselves, which alter water status and affect intercellular spaces.

In summary, as they appear to be associated with established physiological stress responses, the wavelengths identified to be important here appear to be plausible. Further work should look into the application of these wavelengths within practical settings in the field, ideally using cheaper and more easily implemented technology. Combinations of these wavelengths as is commonly done through the use of spectral indices is particularly promising. A preliminary screen of already existing indices yielded unsatisfactory results (data not shown), probably because the conditions under which these indices were developed (crop, age, environment etc.) differ substantially to those of our own experiment. The development of indices that are more specific to the crop and conditions employed here therefore serves as a promising future endeavor.

### Spectral technology for detecting multiple stresses in agriculture

Real world application of spectral technology will undoubtedly involve the detection of not one but several stresses at a time. Here, we have demonstrated that spectral technology does indeed possess the potential to detect and distinguish between multiple stresses, even at pre-symptomatic stages. Similarly, Mahlein et al.^59^ found that while the wavelengths that correlate with disease severity vary among different diseases of sugar beet, reflectance at around 700 nm is strongly correlated to all of the three fungal leaf diseases they investigated.They also found significant spectral differences between healthy and infected plants from the early symptomatic stages onwards. Cotrozzi & Couture^60^ examined the use of hyperspectral spectroscopy for the detection of multiple stresses alone and in combination in lettuce. They found good predictive accuracy while predicting multiple stresses individually but found that as stresses were combined the predictive accuracy was reduced.

Indeed, the detection of multiple stresses using spectral technology is complicated by the fact that several stresses can result in overlapping symptoms and one stress can often be a consequence of another. The stresses applied in our study are relatively varied in their mechanisms in which they negatively affect the plant. Even so, the trees subjected to waterlogging and Metamitron treatment, while often both separable from the control group, did not show distinctly different behaviours from each other in terms of their spectral responses in all but one (2055 nm) of the top 10 most important wavelengths (see Fig. 3).

Using remote sensing technology and looking at more closely related stresses, Zarco-Tejada et al. (2021), nevertheless, did successfully use aircraft-mounted hyperspectral and thermal cameras to distinguish between water deficit stress and two bacterial pathogens (*Xylella fastidiosa, Verticillium dahliae*) that affect the tree-water dynamics in Olive and Almond. Ortiz et al.^61^ reported achieving accuracies of up to 70% in detecting *Fusarium oxysporum* infection and water deficit stress in pre-symptomatic stages on tomatoes (*Solanum lycopersicum *L.) using VIS–NIR spectroscopy from three days after infection. The study showed that reflectance in the range of 510–520 nm and 650–660 nm played a crucial role in detecting *F. oxysporum* stress, especially in the initial stages, while wavelengths of 750 nm and 900 nm were significant for identifying water deficit stress.

Taken all together, the results from this study as well as those already reported in other studies suggest the use of spectral based detection methods does indeed have the potential to be used for the detection multiple stresses in plants, possibly even in the early or, indeed, pre-symptomatic stages. It is important to address the practical application and analysis of stress techniques in real-world settings, considering the challenges of multiple stress occurrences and the need for user-friendly tools. While spectroradiometers may not be farmer-friendly, they serve as a robust choice for initial research. Future research should focus on developing accessible tools for practical and commercial use in agriculture.

Our analysis relies on the first derivative of the reflectance, which may not always have an intuitive interpretation of the results and necessarily requires the data to be continuous (i.e. hyperspectral). This poses significant limitations on the methods by which this data could be generated in the field. Furthermore, it is worth noting that our study was carried out under controlled conditions with a relatively small sample size, using uniform plant material experiencing similar intensities of each stress—somewhat of a simplification of true field conditions. In the field, there would be significantly more environmental variation and interference, and as already mentioned, various stresses often occur simultaneously and at varying intensities. Future studies should explore the effectiveness of spectroscopy for detecting and distinguishing various types of plant stress across diverse plant species and environmental conditions. These studies should consider site variations, including crop variety, soil type, and plant age, and conduct field trials to test and improve the application of early stress diagnosis. Ultimately, this will lead to optimized resource utilization in agriculture.

## Conclusion

In conclusion, our study highlights the effectiveness of using hyperspectral in vivo spectroscopy and machine learning techniques to identify and distinguish between different types of stresses (treatments) in apple trees at the pre-symptomatic stage with high overall accuracy. The reduced models, based on the top 10 wavelengths, provided good prediction performance and yielded insights into potential underlying physiological mechanisms. Our findings suggest that wavelengths related to photosynthesis via pigment functioning (684 nm) or the leaf water (~ 1800–1900 nm) of the plant are important for the correct classification of stresses. Based on our findings, the identified wavelengths hold great potential for the development of accurate and efficient diagnostic tools for the early detection and differentiation of plant stresses, which could ultimately lead to more effective disease management and sustainable agriculture practices.

## Material and methods

### Samples and treatments

To ensure homogeneity among the plants, we utilized 3-year-old, bench-grafted potted apple trees (*Malus x domestica* Borkh.) of the `Golden Delicious´ variety, with a height of approximately 70 cm. The rootstock was M9 and the trees were fertilized once a year (spring) with NPK fertilizer (Nitrophoska Special 12–12–17 (+ 2 + 20). Only healthy plants without any symptoms were selected for use. The plants were acclimatized by transferring them into a greenhouse 14 days prior to the start of the treatments, where the temperature was maintained at 25 °C with 60% relative humidity. To evaluate the effects of both biotic and abiotic stress, three different treatments were conducted: scab (*Venturia inequalis*), waterlogging, and herbicide (Metamitron, Brevis^®^, Adama Italia S.r.l), as well as a control group. Each treatment was replicated 5 times.

Scab was selected as the biotic stress in our experiment as it is the most significant fungal disease in temperate regions^62^. We selected waterlogging as a stress type in our study due to its relevance in flood-prone regions, particularly in the valley bottoms of our study area, where apple orchards are susceptible to submerged conditions caused by frequent floods in spring. This choice allows us to investigate the physiological responses of apple trees to this specific environmental challenge. Given the predicted increase in waterlogging events due to climate change, waterlogging stress becomes increasingly important for orchard management. The anoxic conditions in the root zone are known to significantly impact root metabolism due to anoxia. Metamitron (Brevis^®^) is commonly used in agriculture as both a herbicide (in sugar and fodder beet) and for fruit thinning of apple trees. As a photosynthetic electron transport chain inhibitor, Metamitron reduces photosynthesis and induces stress by disrupting energy metabolism^63^. Due to the specific requirements regarding the onset of stress for each stress type, different exposure times were carefully selected for each treatment.

#### Scab treatment

For the inoculation with apple scab, 5 days before our spectroscopic data collection, we obtained scab-infected leaves from a nearby orchard displaying strong symptoms. The collected leaves were placed in transparent plastic bags, six leaves per bag, and immediately transported to the greenhouse. A branch from our experimental potted trees was sprayed with water until dripping wet. The inside of the plastic bags was also sprayed with approximately 20 mL of water. The bags with the collected leaves were then secured around the wet branch using a wire. After 96 h, the bags with the inoculum were removed. Based on the Mills table and the temperature conditions in the greenhouse, the onset of infection was predicted to happen within 10 h^64^. Successful infection was confirmed by observing dull spots on the leaves of the trees 14-days after the inoculation process (9 days after the spectroscopic measurements were taken).

#### Waterlogging treatment

To expose the plants to waterlogging, each plant was placed in a bucket filled with water until the entire pot and substrate were submerged. This treatment was started 4 days prior to the hyperspectral measurements. We selected a 4-day duration for the waterlogging treatment based on previous studies demonstrating significant physiological responses within a few days of exposure^65^.

#### Herbicide (Metamitron)

For the herbicide treatment, plants were treated with Brevis^®^ (Adama Italia S.r.l.; active agent: Metamitron) 24 h before the hyperspectral measurements. A spray bottle was used to apply 20 mL of the Metamitron solution, to each tree (replicate). The solution had a concentration of 0.93 g/L, which is the recommended concentration for fruit thinning of apple trees. Taking into account previous research indicating substantial physiological responses, such as photosynthesis inhibition, within a 24-h timeframe of herbicide exposure, we selected a 24-h interval for the application of Metamitron in the herbicide treatment^66^.

#### Hyperspectral measurements

We used a portable spectroradiometer (HR1024i, Spectra Vista 126 Corporation, Poughkeepsie, NY, USA) to measure the hyperspectral reflectance of the leaves with an external fibre-optic and leaf clip assembly (LC-127 RP PRO, Spectra Vista Corporation). The device possessed a wavelength range from 350 to 2500 nm with a sampling interval of 1 nm. From each plant, 10 technical replicates were taken, which makes a total of 50 measurements per treatment. The measurements were taken on June 1st, 2021, after the onset of full sunlight (9:00 am) to ensure the occurrence of photosynthesis and physiological activity in the plants.

#### Data processing and model training

The data processing and analysis was conducted using R statistical language^67^. Raw data was screened for outliers through shape anomaly analysis of the spectral signature and boxplot outlier detection (points outside of the upper and lower whiskers). The coefficient of variation (CV = standard deviation/mean), a statistical measure of relative variability, was used as a metric to quantify the variations in our measured spectral data for each treatment.

Noise-reduction by curve smoothing as well as the first Savitsky-Golay derivative of the original spectral data was generated from the raw data using the hsdar package in R^68^.The transformation of the data into its first derivative removes additive effects and often improves model performance (and as was indeed the case here)^69^. The data set was divided into a training set and a test set with a split ratio of 80:20, ensuring that the testing data was not used in the training process. The data consisted of 168 samples and 2036 features (variables), with a spectral range from 365 to 2400 nm and a sampling interval of 1 nm.

We classified our data according to two labelling systems:

Firstly, for the purpose of distinguishing and classifying specific stress types, we assigned their specific stress labels i.e., four distinct classes: control, scab, metamitron, and water logging. Secondly, to train models to differentiate between “healthy” and “stressed” conditions, we employed general stress or health status labels. All trees experiencing any of the three of the stress treatments were assigned to a “stressed” class. While those under the control treatment was labelled as “healthy”, resulting in two classes.

Principal Component Analysis (PCA) is a popular unsupervised machine learning technique used to reduce the number of dimensions and identify features that explain most of the variance within the independent variables^70^. The PCA was applied in this study to: (a) determine if the treatments cluster at a first glance, (b) identify the dimensions (PCs) that show the best clustering and separation of treatments, and (c) identify the important variables that contribute to the clustering and differentiation. The PCA was carried out using the factorminer and factoextra packages in R^71,72^.

We trained three classification models: support vector machine (SVM), random forest and partial least squares-discriminant analysis (PLS-DA). These models were chosen for their ability to handle high dimensional data and their performance in previous studies. The SVM algorithm utilizes the concept of maximal margin classifiers and kernel functions to separate the data into different classes. When training multiclass classification SVMs, a ‘one-against-one’ approach was used. The Random Forest algorithm is an ensemble method that constructs a multitude of decision trees and outputs the class that is the mode of the classes output by individual trees. PLS, similar to PCA, is a data summarization technique that finds orthogonal linear combinations of variables (latent variables) that best describe the relationships between the predictors and response variables^63^. Discriminant analysis is then performed on the latent variables to find linear combinations (i.e., decision boundaries) by maximizing the ratio of between group-variance to within group variance^73^. Each model was trained and evaluated on the same dataset to compare their performance.

For the SVM model, a linear kernel was used by passing the *ksvm* (method = svmLinear) function provided by the R package Kernlab to the *caret::train* function^74,75^. The training data were pre-processed by centering and scaling the predictors. Cross-validation was performed using tenfold with 3 repeats to tune the regularization hyperparameter ‘C’ (Cost of Constraints). The optimized C-value (0.001) was chosen from a range between 0.001 and 5 and a ‘tuneLength’ of 10.

The Random Forest model used the ranger implementation with 500 trees and importance measure. Hyperparameters ‘mtry’ (the number of randomly selected predictors), ‘splitrule’ (the splitting rule) and ‘min.node.size’ (the minimum node size) were tuned using a grid search—the optimized values were 225, “gini” and 1 respectively. Tenfold cross-validation with 3 repeats was used for model validation.

In order to optimize the performance of the PLS model, we used cross-validation to tune the number of latent variables in the model. The number of latent variables was varied up to 12 and the optimal value was chosen using tenfold cross-validation, repeated 3 times.

We used the trained Random Forest model to identify important variables (wavelengths) because its architecture allows for the efficient, reliable and interpretable extraction of feature importance^76^. We then trained “reduced” models using the subsets of variables identified by the model. The feature importance was obtained from the Random Forest models as the impurity corrected Gini importance^76^. To obtain class-specific variable importance, ROC (receiver operating characteristic) curve analysis of the Random Forest model was used where each predictor (i.e. wavelength band) is assessed individually based how well the classes are ranked according to that predictor (the reflectance in that wavelength band) calculated as the area under the curve (AUC) Pairwise comparisons between each class are performed and the importance of a given predictor for a class is reported as the maximum area AUC out of each of these pairwise comparisons. To validate the importance of the selected features, the first derivative reflectances at each of these wavelengths were subsequently subjected to Analysis of Variance (ANOVA) followed by Tukey’s honest significant difference post-hoc tests.

### Statement on the collection of plant material

The plant materials used in this study were sourced from controlled cultivation and all collection were made in accordance with institutional, national and international guidelines for the collection of wild plants.

### Supplementary Information

Supplementary Figures.

## Data Availability

The datasets used and/or analysed during the current study available from the corresponding author on reasonable request.
