# Supplementary material for: In vivo spectroscopy and machine learning for the early detection and classification of different stresses in apple trees
Source: Sci Rep. 2023 Sep 22;13:15857. doi: 10.1038/s41598-023-42428-z (PMC10517117; doi:10.1038/s41598-023-42428-z)
Supplement: Supplementary file 1 — Supplementary Figures. [file 41598_2023_42428_MOESM1_ESM.docx]

Supplementary INFORMATION

of the article:
**Early detection of multiple stresses on apple trees using in-vivo leaf spectroscopy and machine learning: revealing the importance of leaf water-related wavelengths.**

DOI:

Authors:

Ulrich E. Prechsl Abraham Mejia-Aguilar^2^ Cameron B. Cullinan^1,3^

^1^ Laimburg Research Centre, Laimburg 6, Auer/Ora, BZ 39040, South Tyrol, Italy

^2^Eurac Research, Drususallee 1/Viale Druso 1, IT-39100 Bolzano, South Tyrol, Italy

^3^Faculty of Agricultural, Environmental and Food Sciences, Free University of Bolzano, Piazza Università 1, BZ 39100, South Tyrol, Italy

*Corresponding author: uli.prechsl@gmail.com


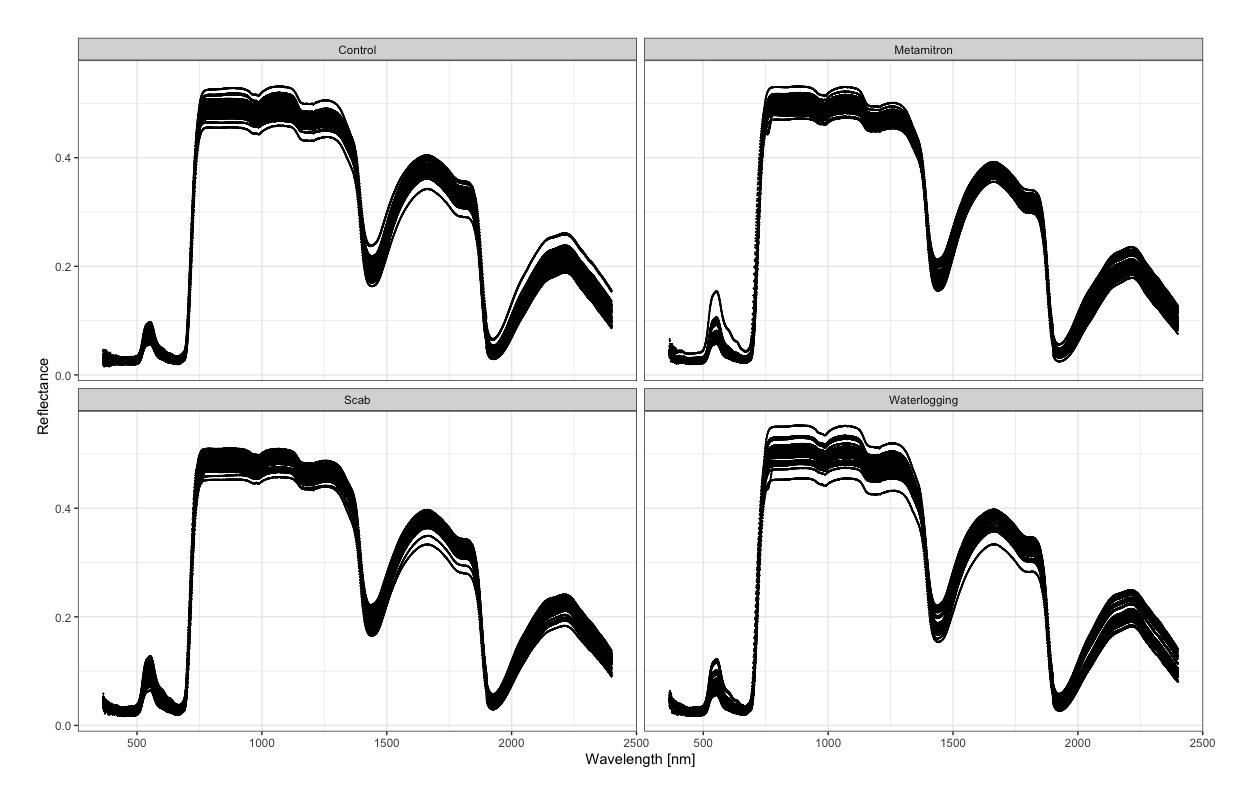


**Figure S1:** Spectral reflectance of 3-year-old Golden Delicious apple trees that were exposed to different types of stress (herbicide Metamitron, apple scab and waterlogging, n=5). Each line represents a technical replicate (n=10) measured at a different leaf.


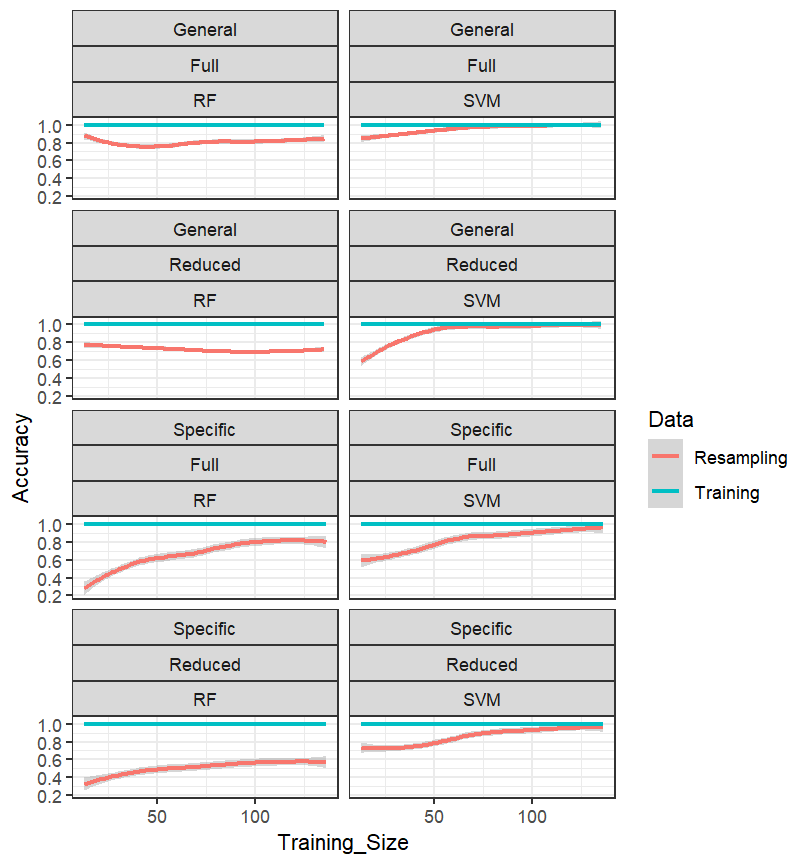


**Figure S2**: Learning curves for full spectra models (Full) and models using selected wavelengths (Reduced) for the detection of stress in general (General) and each individual stress (Specific) for the Random Forest (RF) and Support Vector Machine (SVM) models. Training and resampled cross-validation accuracies as a function of resampled dataset size are shown by green and red lines, respectively.


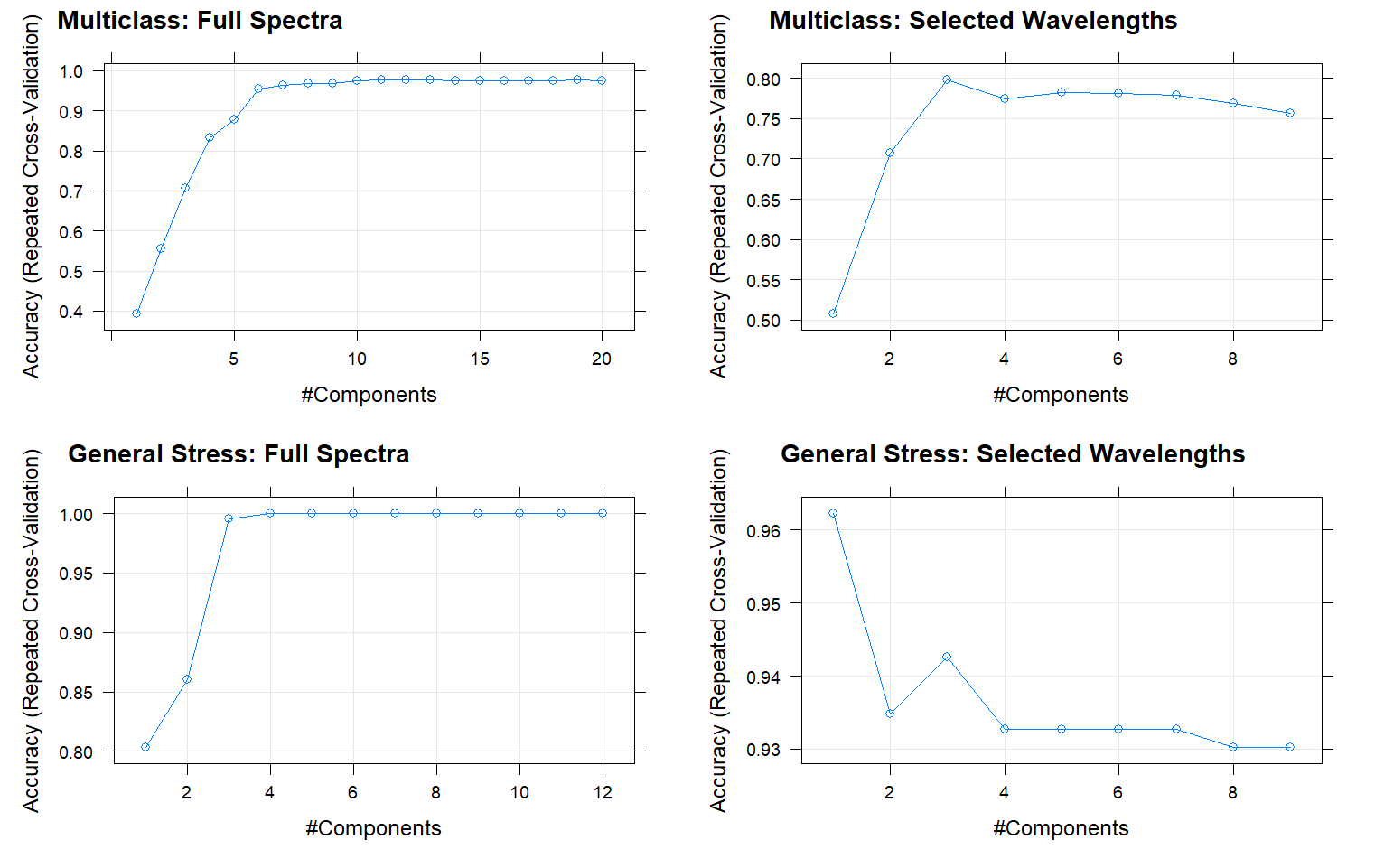


**Figure S3**: Validation curves for PLS models using full spectra or selected wavelengths for the detection of stress in general or each individual stress (multiclass models).
